# The p16/ki-67 assay is a safe, effective and rapid approach to triage women with mild cervical lesions

**DOI:** 10.1371/journal.pone.0253045

**Published:** 2021-06-11

**Authors:** Maria Magkana, Panagiota Mentzelopoulou, Ekaterini Magkana, Andreas Pampanos, Georgios Daskalakis, Ekaterini Domali, Alexandros Rodolakis, Kalliopi Pappa

**Affiliations:** 1 School of Medicine, National and Kapodistrian University of Athens, Athens, Greece; 2 Department of Cytology, "Alexandra" General Hospital, Athens, Greece; 3 Department of Genetics, "Alexandra" General Hospital, Athens, Greece; 4 1^st^ Department of Obstetrics and Gynecology, "Alexandra" Hospital, National and Kapodistrian University of Athens, Athens, Greece; 5 Cell and Gene Therapy Laboratory, Centre of Basic Research II, Biomedical Research Foundation of the Academy of Athens (BRFAA), Athens, Greece; Ruđer Bošković Institute, CROATIA

## Abstract

**Objective:**

The aim of this study was to evaluate the diagnostic accuracy and efficiency of p16/ki-67 dual stain in the identification of CIN2+ lesions, in Greek women with ASCUS or LSIL cytology.

**Methods:**

A total of 200 women, 20 to 60 years old, were enrolled in the study. All samples were cytologically evaluated and performed for p16/ki-67 and high-risk HPV (HR-HPV) test. All patients were referred to colposcopy for biopsy and histological evaluation. Three cervical cancer (CC) screening strategies were designed and the total direct medical costs of the procedures during our clinical trial were evaluated, from a healthcare perspective.

**Results:**

HPV 16 as expected was the most common HR-HPV type followed by HPV 31 and HPV 51. The risk for CIN2+ was significantly higher in HPV 16/18 positive cases. p16/ki-67 demonstrated a high sensitivity for CIN2+ identification in both ASCUS and LSIL groups (90.4% and 95%, respectively). HR-HPV test with sensitivity 52.3% and 65.5%, as well as colposcopy with sensitivity 14.3% and 36% respectively in ASCUS and LSIL group, showed inferior results compared to p16/ki-67. The specificity of p16/ki-67 for ASCUS and LSIL was 97.2% and 95.2% respectively, inferior only to colposcopy: 100% and 100%, lacking however statistical significance. HR-HPV test instead, presented the lowest specificity: 76.4% and 71.4% respectively in comparison to the other two methods. From a healthcare perspective, the costs and benefits of the tests implementation for the annual screening and triaging, in three CC screening strategies, were also calculated and discussed.

**Conclusions:**

The results of the study indicate that p16/ki-67 is a safe and rapid assay that could be used to detect CIN2+ among women with mild cervical lesions, presenting both high sensitivity and specificity and could minimize the psychological and economic burden of HPV screening.

## Introduction

Cervical cancer (CC) is the fourth most common malignancy in women globally. It has been established as a great disease model for prolepsis, as it has been significantly reduced during the past decades due to the population-based screening programs and vaccination [1]. The main screening tool for CC has been for many years cytology (Pap test), which contributed to the reduction of the disease in women between 35 to 64 years old, by at least 80% [2–4]. Nowadays, the American Society for Colposcopy and Cervical Pathology has approved three primary CC screening approaches for women between 21 and 65 years old: the cytology, the high-risk human papillomavirus (HR-HPV) DNA testing and the co-testing (cytology plus HPV testing) [5–7]. According to many studies, the Pap test has limitations, with the most striking being its low sensitivity [5, 8]. In Greece though, cytology remains still the primary screening tool and the cost is fully covered by the National Health Organization System. However, the population compliance is not sufficient, e.g. CC screening coverage in 2018 was 68.8% [9].

Furthermore, besides the screening approach, triage tests are needed to identify among HPV positive women, those that are at a higher risk and should be referred to colposcopy and further diagnostic evaluation. HPV testing is been proven to be a useful triage tool for minor cytological abnormalities, such as atypical squamous cells of undetermined significance (ASCUS) when we screen women over 30 years old [7, 10].

On the other hand, HPV testing as primary screening presents high sensitivity but low specificity, resulting in an increased number of HPV positive patients who should be referred to colposcopy. That type of management increases the burden in colposcopy clinics and the risk of overtreatment. Thus, an optimal triage strategy which combines both high sensitivity and specificity, is needed to distinct the majority of women at low risk of developing cervical cancer and identify those at a higher risk, who should be referred to colposcopy for further management or treatment [11, 12]. Therefore, some sufficient biomarkers have been proposed such as HPV E6/E7 mRNA test and p16^INK4a^ protein [11, 13–16].

The aim of this study was to evaluate the diagnostic ability of p16/ki-67 dual stain in a homogenous Greek population, in order to identify the optimal management strategy of HR-HPV positive women with ASCUS or low-grade squamous intraepithelial lesion (LSIL) cytology as part of an organized cervical cancer screening setting.

## Materials and methods

### Study population

The present study was conducted in the 1^st^ Department of Obstetrics and Gynecology, "Alexandra" Hospital, National and Kapodistrian University of Athens, in Greece. A total of 200 Greek women aged from 20 to 60 years old with ASCUS/ LSIL cytology, HR-HPV positive and with histological findings CIN≥ 1 (cervical intraepithelial neoplasia), were selected as a subpopulation of a larger study. These women attended the clinic from March 2018 through April 2019 for their routine cytology-based cervical cancer screening and after being thoroughly informed participated in the study. Liquid-based cytology (LBC) samples were collected and after HPV testing and p16/ki-67 dual stain were performed, women were referred to colposcopy for evaluation and cervical biopsies. Three cases were excluded as invalid cytology and one as invalid p16/ki-67 due to low cell count.

The exclusion criteria of the current study were HR-HPV negativity or biopsy result of CIN0, treatment for CIN, prior hysterectomy and pregnancy. Written informed consent, medical history and demographic information were obtained from all the 196 enrolled subjects (Table 1). Medical history involved previous cytology tests, HPV vaccination and other vaginal infections. Demographic variables included: age, sexual and reproductive behavior and smoking status. The study was approved by the Ethics Committee of “Alexandra” Hospital.

**Table 1:** Demographic variables and health status of our population.

Demographic characteristics	All (N = 196)	
	N	(%)^a^
**Age (years)**		
< 30 years old	53	27.0
≥ 30 years old	143	73.0
**No. of lifetime sexual partners**		
1	16	8.2
2–4	70	35.7
≥ 5	110	56.1
**No. of full-term pregnancies**		
Nulliparous	94	48.0
1–2	97	49.5
≥ 3	5	2.5
**Smoking status**		
Non smoker	95	48.5
Smoker	101	51.5
**Health status**		
**Cytology tests the previous years**		
Yes	178	90.8
No	18	9.2
**HPV vaccination**		
Yes	25	12.8
No	171	87.2
**Other vaginal infections**		
Yes	51	26.0
No	145	74.0

N, number; Nulliparous, no pregnancies.

^a^ Values are presented as percentage (%).

### Cytology—Procedure

Liquid-based cytology samples were stored in PreservCyt solution (Thinprep, Hologic, U.S.A) vials according to the manufacturer’s instructions. A first slide was prepared using Thinprep T2000 processor system (Hologic, U.S.A) for cytological evaluation in accordance to the 2014 Bethesda system [17]. For this study we enrolled cases which presented ASCUS and LSIL cytology. A second slide was used for the p16/ki-67 dual stain (DS) assay. From each Thinprep vial, HPV testing was processed by two methods: Cobas^**®**^ HPV test and CLART^®^ HPV3 genotyping assay.

### HPV testing

#### Cobas^®^ HPV test

Qualitative in vitro HPV genotyping analysis was performed using Cobas^®^ HPV test (Roche Molecular Systems, Inc., Branchburg, NJ) which provides individual results for HPV 16 and HPV 18 genotypes and pooled results for the rest 12 HR-HPV types: 31, 33, 35, 39, 45, 51, 52, 56, 58, 59, 66 and 68 in a single analysis. Briefly, target DNA primers amplify a sequence of approximately 200 nucleotides within the L1 region of the HPV genome of the sample with the use of polymerase chain reaction (PCR) to detect the 14 high-risk HPV types [18]. The whole process was performed in the Cobas^®^ 4800 fully automated System.

#### CLART ^®^ HPV3 assay

HPV genotyping of the 12 HR-HPV types, that were not evaluated separately with Cobas technology, was performed with the CLART ^®^ HPV3 kit (Genomica S.A.U, Madrid, Spain), an in vitro diagnostic kit that detects 49 HPV genotypes including High Risk (16, 18, 31, 33, 35, 39, 45, 51, 52, 56, 58, 59, 66, 68), Prob. High Risk (26, 53, 73, 82), Low Risk (6, 11, 40, 42, 43, 44, 54, 61, 62, 70, 71, 72, 81, 83, 84, 85, 89) and genotypes of undetermined risk (for research only): 34, 64, 67, 69, 74, 86, 87, 97, 101, 102, 103, 106, 150, 151. Detection is based on CLART^®^ technology which includes PCR amplification of a 450bp fragment within the L1 viral region using type-specific probes. The visualization of the amplified products is performed in a low-density microarray platform and interpreted in an automatic Genomica’s reader (CAR^®^ or Clinical array reader) [19]. For the purposes of the current study, we involved cases positive for the 14 HR-HPV genotypes.

### p16/ki-67 dual stain (CINtec^®^ PLUS Cytology kit)

The CINtec^®^ PLUS Cytology Kit is an immunocytochemistry assay for the qualitative detection of p16^INK4a^ and ki-67 proteins in cervical cytology preparations. The proteins are detected using a ready-to-use cocktail of primary monoclonal antibodies: human p16^INK4a^ protein (clone E6H4^™^) and primary recombinant rabbit antibody directed against human ki-67 protein (clone 274–11 AC3) [20]. The slides for p16/ki-67 dual stain were performed using CINtec^®^ PLUS kit (Roche mtm laboratories AG, Mannheim, Germany) on an automated slide-stainer platform: Ventana BenchMark XT Slide Stainer.

**Fig 1:**
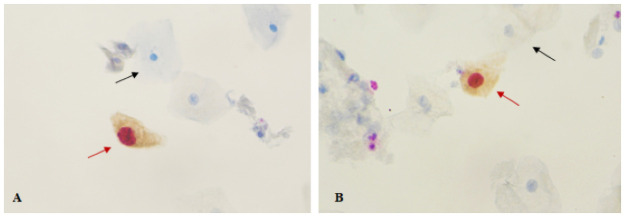
Examples of p16/ki-67 dual stained cells.

All slides were evaluated by two experienced cytopathologists unaware of histology and HPV testing results. A case was interpreted as positive if one or more cervical epithelial cells were dual stained for both p16^INK4a^ (brown cytoplasmic stain) and ki-67 (red nuclear stain) regardless of their morphologic appearance. Negativity was established when no synchronous p16/ki-67 dual staining in the same cell was observed, in accordance to the manufacturer’s criteria and instructions [21]. In Fig 1A and 1B below, examples of p16/ki-67 dual stained cells in cases of ASCUS and LSIL cytology are presented. A case was excluded or scored as invalid if no p16 ^INK4a^ and/or ki-67staining was visible or if there were less than 5,000 squamous cells in the slide [21].

p16/ki-67 dual-stained examples (×40 magnification) in cases of (A) atypical squamous cells of undetermined significance (ASCUS), (B) low-grade squamous intraepithelial lesion (LSIL) cytology. Positive p16/ki-67 dual-stained cells with a brown cytoplasmic stain for p16^INK4a^ overexpression and a dark red nuclear stain for ki-67 expression within the same cell (red arrow); unaffected cervical epithelial cells without p16/ki-67 staining (black arrow).

### Colposcopy and histology

The population enrolled in our study, underwent colposcopic evaluation of the cervix and the colposcopic findings were reported according to the ASCCP Terminology for Colposcopic Practice [22]. If no visible lesion was found, the colposcopic impression was classified as ‘Negative’, whereas positivity was then reported as ‘LSIL’ (low-grade abnormalities) or ‘HSIL’ (high-grade abnormalities). Biopsy from visible cervical lesions was performed by qualified, trained physicians. Histopathologic review of the cervical biopsies was performed from specialized pathologists blinded to all laboratory results. Cervical histopathological diagnoses included CIN≥ 1 (CIN1, CIN2, CIN3, or cancer).

### Screening strategies and costs data

We designed and evaluated three different CC screening strategies based on the current algorithms used: Cytology (LBC), HPV test with 16/18 genotyping, Co-testing (Cytology & HPV test with 16/18 genotyping) with the addition of p16/ki-67 dual stain as a triage strategy.

The costs of the lab-tests for screening (Cytology, HPV test with 16/18 genotyping, p16/ki-67 dual stain) and the diagnostic methods (Colposcopy, Cervical biopsy and office visit to specialist) presented in Table 2 below were evaluated from a healthcare perspective, in Public Healthcare System or Private Practice. In Public Healthcare System, cost inputs were obtained in 2019, from the official price list of the National Organization for the Provision of Health Services (EOPYY) in Greece. In the Private sector, costs were based on 2019 price lists of two different Private Hospitals in Greece (Private Practice 1&2) and the average costs were evaluated. In Public Healthcare System, the cost of p16/ki-67 dual stain would be covered by the healthcare system when used in practice. Therefore, its cost was assumed to be the same as the Private Practice’s price.

**Table 2:** Costs of the lab-tests and diagnostic methods for HPV screening.

	Expenditures per procedure (€)^a^			
Lab-Test	Private Practice 1	Private Practice 2	Average cost in Private Practice ^b^	Public Healthcare System^c^
HPV test with 16/18 genotyping	140,00	135,00	137,50	80,00
Cytology test	60,00	55,00	57,50	13,32
p16/ki-67 dual stain	60,00	60,00	60,00	Private practice (60,00)*
**Diagnostic methods**				
Colposcopy	150,00	150,00	150,00	17,61
Cervical Biopsy	30,00	55,00	42,50	21,13
Office visit to specialist	100,00	200,00	150,00	0

Cytology, LBC, Liquid-based cytology.

^a^ Values are presented in Euros (€).

^b^ Costs obtained from the price lists of Private practice 1&2.

^c^ Costs derived from EOPYY.

*The cost of p16/ki-67 dual stain was assumed to be the same as the Private Practice’s price.

In each CC screening strategy we included the direct medical costs of the procedures per screened woman from a healthcare perspective. Specifically, direct medical costs consist of screening tests used (Cytology, HPV test with 16/18 genotyping, p16/ki-67) and diagnostic methods (Colposcopy and Cervical biopsy) plus office visit (physician’s time for patient’s evaluation). The costs for the annual follow-up screening tests as well as any treatment needed for CIN were not included.

#### First strategy: Cytology as primary screening test (Fig 2)

**Fig 2:**
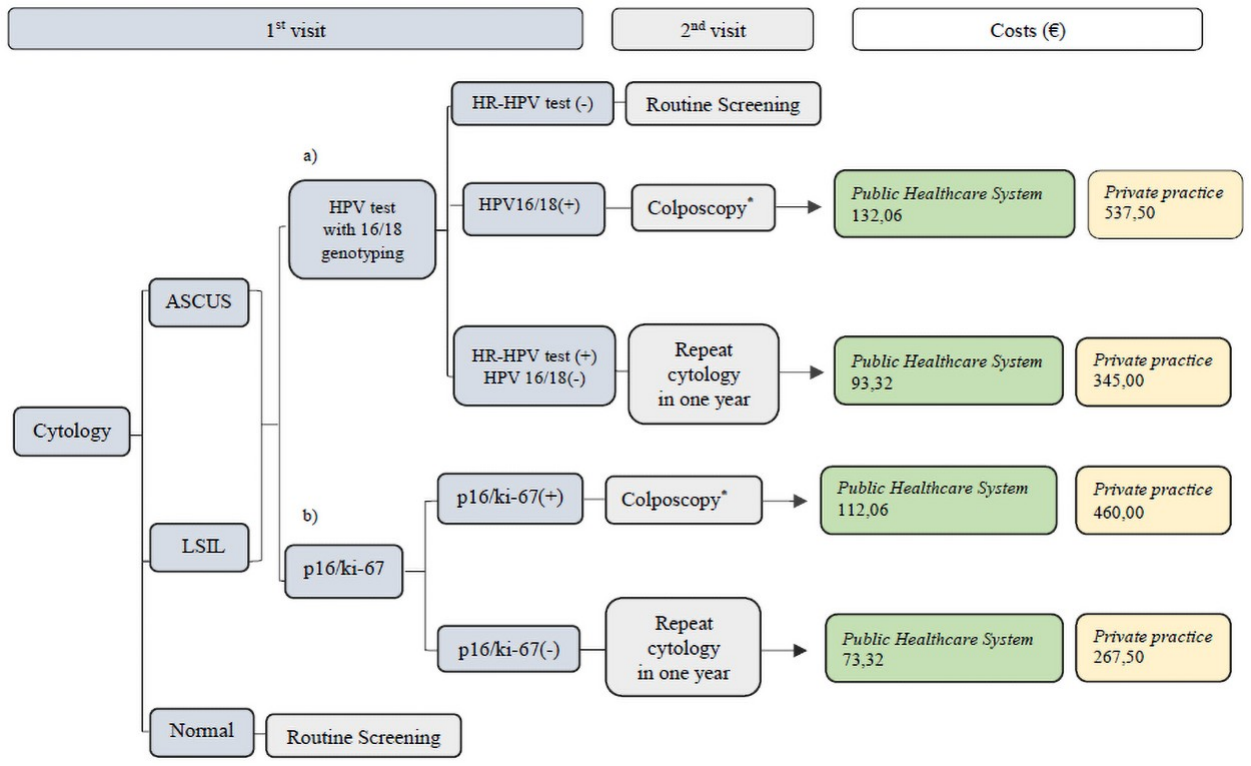
First strategy: Cytology as primary screening test. Triage with a) HPV test with 16/18 genotyping and b) p16/ki-67 dual stain. Cytology, LBC; Costs in Euros (€), direct medical costs (screening tests, colposcopy with biopsy and office visit) per woman screened; costs for annual follow-ups and treatments for CIN were not included. *Colposcopy with biopsy.

Women with Normal cytology return to Routine Screening.

Women with Cytology ≥ ASCUS:

**a)** Triage with HPV 16/18 genotyping.
Women with HR-HPV test (-) return to Routine Screening, those with HPV16/18(+) are referred to colposcopy, whereas women with HR-HPV test (+) & HPV16/18(-) repeat cytology in one year.
**b)** Triage with p16/ki-67 dual stain.
Women with p16/ki-67(+) results are referred to colposcopy while those with p16/ki-67(-) repeat cytology in one year.

#### Second strategy: HPV test as primary screening test (Fig 3)

**Fig 3:**
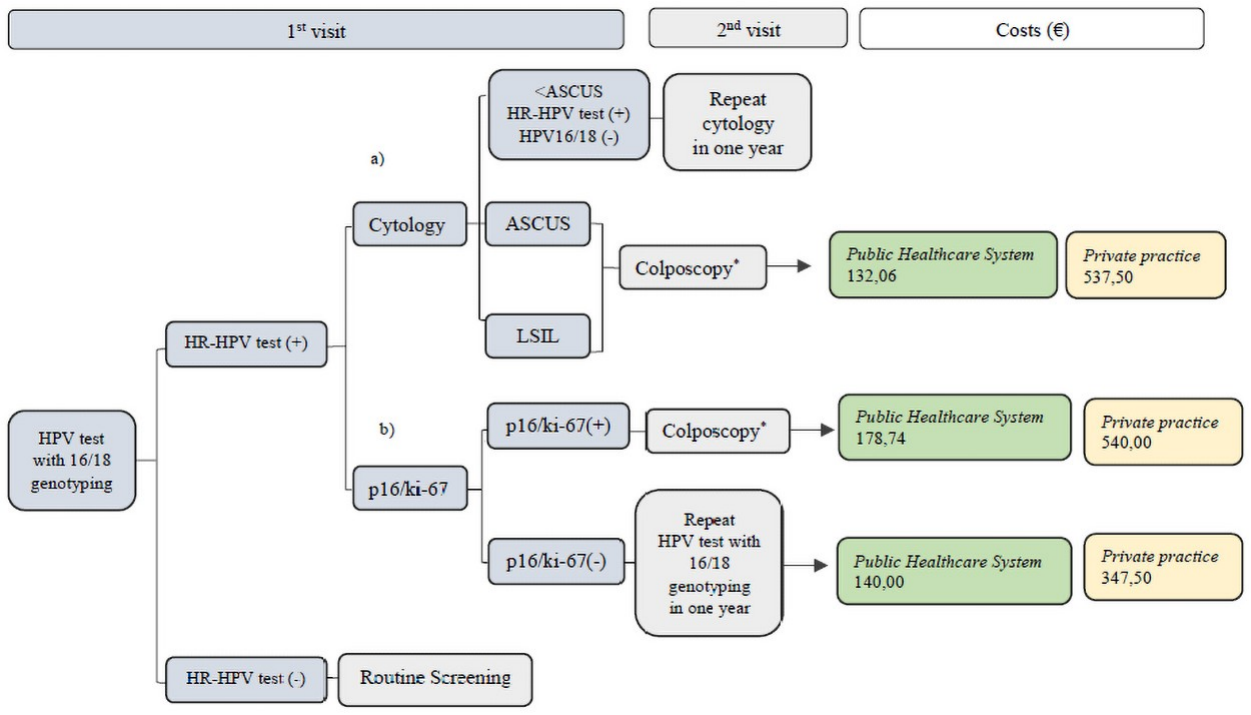
Second strategy: HPV test as primary screening test with 16/18 genotyping. Triage with a) Cytology b) p16/ki-67dual stain. Cytology, LBC; Costs in Euros (€), direct medical costs (screening tests, colposcopy with biopsy and office visit) per woman screened; costs for annual follow-ups and treatments for CIN were not included. *Colposcopy with biopsy.

Women with HR-HPV test (-) return to Routine Screening.

Women with HR-HPV test (+):

**a)** Triage with cytology.
Women with HR-HPV test (+) & HPV16/18(-) with cytology < ASCUS, repeat cytology in one year.
Women with an HR-HPV test (+) and cytology ≥ ASCUS are referred to colposcopy.
**b)** Triage with p16/ki-67.
Women with p16/ki-67(+) are referred to colposcopy, while those with p16/ki-67(-) are re-tested with HPV 16/18 genotyping in one year.

#### Third strategy: Co-testing (Cytology & HPV test with 16/18 genotyping) as primary screening (Fig 4)

**Fig 4:**
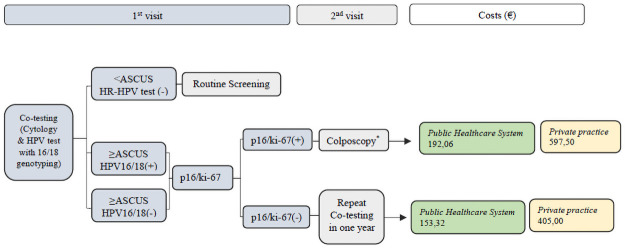
Third strategy: Co-testing as primary screening. Triage with p16/ki-67 dual stain. Cytology, LBC; Costs in Euros (€), direct medical costs (screening tests, colposcopy with biopsy and office visit) per woman screened; costs for annual follow-ups and treatments for CIN were not included. *Colposcopy with biopsy.

Women with Cytology <ASCUS and HR-HPV test (-) return to Routine Screening.

Women with Cytology ≥ ASCUS and HR-HPV test (+) are triaged with p16/ki-67 dual stain. When p16/ki-67(+) the patients are referred to colposcopy and those with p16/ki-67(-) repeat co-testing in one year.

### Statistical analysis

Data were expressed as mean ± SD for continuous variables and as percentages for categorical data. The Kolmogorov—Smirnov test was utilized for normality analysis of the parameters. To evaluate the risk of CIN2+ lesions, according to HPV genotype, the Chi-square test (χ^2^) and Odds Ratio (O.R.) with 95% Confidence Interval (CI) were calculated. Diagnostic accuracies of HR-HPV test, Colposcopy and p16/ki-67 method for the detection of CIN2+ were evaluated using Sensitivity, Specificity, Positive Predictive Value (PPV) and Negative Predictive Value (NPV). Comparison between them was performed using the Paired Samples t-test. A Receiver Operating Curve (ROC) analysis was conducted to compare the prognostic abilities of HR-HPV test and p16/ki-67 for the detection of CIN2+ calculating the respective areas under the curve (AUC) with 95% CI, using the maximum likelihood estimation method, which has the advantage of being free of assumption about the Gaussian distribution of underlying variables. All tests were two-sided and statistical significance was set at p < 0.05. All statistical analyses were conducted using SPSS vr 21.00 (IBM Corporation, Somers, NY, USA).

## Results

A total of 200 liquid-based cytology samples with CIN ≥ 1 on histological evaluation and HR-HPV (+) were analyzed. Among those, 93 cases had ASCUS cytology, 103 had LSIL and 4 cases were excluded as invalid due to low cellularity. The mean age of all 196 finally enrolled women was 37.5 years old, with participants < 30 years old (27%) and ≥ 30 years old (73%). Only a small percent (12.8%) of the women were HPV vaccinated, the majority (90.8%) had a pap test performed during the previous years and 26.0% had other vaginal infections. Most women (91.8%) had over 2 sexual partners during their lifetime and approximately half the population was nulliparous. Smoking habits of our population were equally distributed (Table 1).

**Fig 5:**
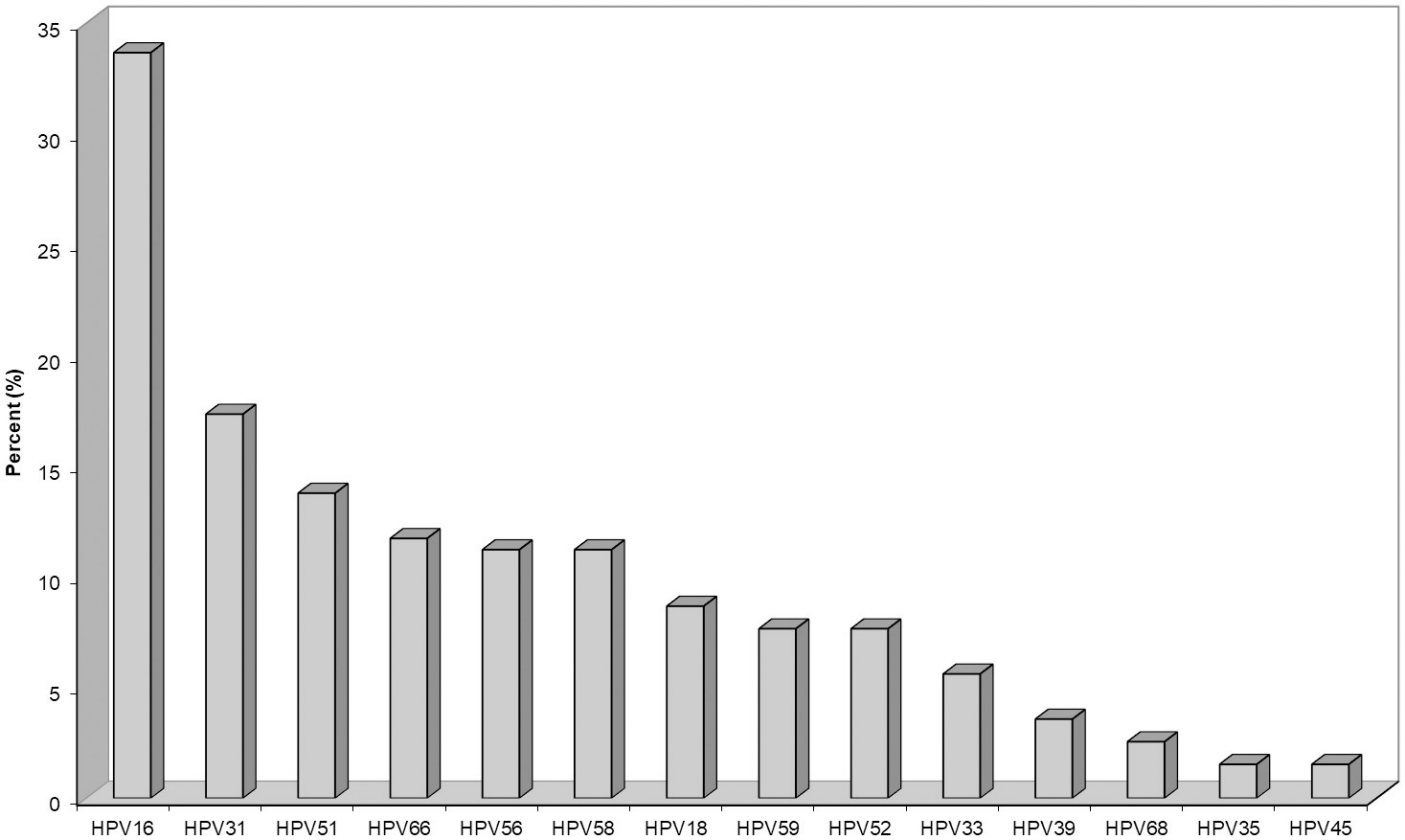
HR-HPV genotypes distribution in the study population. The bars represent a single or multiple type infection, whilst in multiple types the case is classified according to the highest risk type for its constituents.

All patients were selected to be HR-HPV positive and the HR-HPV test results were classified as HPV 16/18 (+) or HPV 16/18 (-) when no HPV 16 or HPV 18 was detected. In our population, 80/196 (40.8%) cases were HPV 16/18 positive and 116/196 (59.2%) had some other of the 12 HR-HPV types. In cases of multiple infections, patients were classified according to the highest risk type for its constituents. For example, if a case distributed both HPV 16 (+) and HPV 31 (+), it was then classified into the HPV 16/18 (+) group. The percentages of HR-HPV genotypes in our study are presented in Fig 5.

HPV 16 was the most frequent high-risk type in 66 out of 196 cases (33.7%) followed by HPV 31 (17.3%) and HPV 51 (13.8%). The HPV 56, HPV 58 and HPV 66 distributed similar percentages (11.2%–11.7%), followed by HPV 18 (8.7%). The HPV 52 and HPV 59 presented a percentage of 7.7% and the rest HR-HPV types: 33, 35, 39, 45, 68 presented lower frequencies.

In order to evaluate the risk of CIN2+ according to HPV genotype, patients were sorted into fourteen groups depending on HR-HPV genotype’s positivity as shown in Table 3. Among patients with HPV16 (+) or HPV18 (+), either with single or multiple infections, the odds ratio for CIN2+ lesions were 3.2 (95% CI 1.74–6.00) and 5.2 (95% CI 1.62–16.53) respectively. These results are statistically significant higher compared with the odds ratio of the rest 12 HR-HPV genotypes.

**Table 3:** Odds ratio for CIN2+ histological diagnosis for each HR-HPV genotype.

HR-HPV type positivity ^a^	% CIN2+ ^b^	Odds ratio ^c^	95% CI ^d^	p-value ^e^
HPV 16 (+)	60.6%	3.22	1.74–6.00	<0.001
HPV 18 (+)	76.5%	5.18	1.62–16.53	0.004
HPV 31 (+)	38.2%	0.83	0.39–1.78	0.705
HPV 33 (+)	63.6%	2.56	0.73–9.08	0.207
HPV 35 (+)	66.7%	2.82	0.25–31.69	0.573
HPV 39 (+)	14.3%	0.22	0.03–1.88	0.242
HPV 45 (+)	0%	---	---	0.266
HPV 51 (+)	29.6%	0.54	0.22–1.30	0.209
HPV 52 (+)	40.0%	0.92	0.32–2.69	1.000
HPV 56 (+)	27.3%	0.48	0.18–1.29	0.172
HPV 58 (+)	41.0%	0.96	0.39–2.36	1.000
HPV 59 (+)	26.7%	0.48	0.15–1.56	0.281
HPV 66 (+)	43.5%	1.08	0.45–2.60	1.000
HPV 68 (+)	20.0%	0.34	0.04–3.09	0.402

HR-HPV, High-risk HPV; CIN, cervical intraepithelial neoplasia; CIN 2+ includes CIN2 or worse lesions.

^a^ Multiple infection cases were not excluded.

^b^ The risk of CIN2+ cases (pathology diagnosis) was presented according to HR-HPV genotype.

^c^ Odds ratio HPV type (positive vs negative) for CIN2+.

^d^ Values are presented as percentages with 95% confidence intervals (CI).

^e^ Statistical significance was set at p < 0.05.

Among the 93 ASCUS samples presented in Table 4, 28 were HPV 16/18 positive with 11/28 (39.3%) CIN2+ and 17/28 (60.7%) CIN1. All ASCUS cases were additionally tested for p16/ki-67 dual staining and 21 cases were p16/ki-67 positive of which 19 (90.5%) were histological CIN2+.

**Table 4:** Correlation of HPV 16/18, p16/ki-67, colposcopy and combination of HPV 16/18/ p16/ki-67 with biopsy results in our population study.

Cytology		CIN1	CIN2+
**ASCUS (N = 93)**		**(N = 72)**	**(N = 21)**
HPV 16/18 (+)		17 (60.7%)	11 (39.3%)
HPV 16/18 (-)		55 (84.6%)	10 (15.4%)
p16/ki-67 (+)		2 (9.5%)	19 (90.5%)
HPV 16/18 (-) / p16/ki-67 (-)		53 (96.4%)	2 (3.6%)
HPV 16/18 (-) / p16/ki-67 (+)		2 (20%)	8 (80%)
HPV 16/18 (+) / p16/ki-67 (-)		17 (100%)	0 (0%)
HPV 16/18 (+) / p16/ki-67 (+)		0 (0%)	11(100%)
**Colposcopy**	Negative	14 (82.4%)	3 (17.6%)
	LSIL	58 (79.5%)	15 (20.5%)
	HSIL	0 (0%)	3 (100%)
**LSIL (N = 103)**		**(N = 42)**	**(N = 61)**
HPV 16/18 (+)		12 (23.1%)	40 (76.9%)
HPV 16/18 (-)		30 (58.8%)	21 (41.2%)
p16/ki-67 (+)		2 (3.3%)	58 (96.7%)
HPV 16/18 (-) / p16/ki-67 (-)		28 (100%)	0 (0%)
HPV 16/18 (-) / p16/ki-67 (+)		2 (8.7%)	21 (91.3%)
HPV 16/18 (+) / p16/ki-67 (-)		12 (80%)	3 (20%)
HPV 16/18 (+) / p16/ki-67 (+)		0 (0%)	37 (100%)
**Colposcopy**	Negative	6 (85.7%)	1 (14.3%)
	LSIL	36 (48.6%)	38 (51.4%)
	HSIL	0 (0%)	22 (100%)

N, number; ASCUS, Atypical squamous cells of undetermined significance; LSIL, Low-grade squamous intraepithelial lesion; CIN, cervical intraepithelial neoplasia; CIN 2+ includes CIN2 or worse lesions; Colposcopy, Negative (Normal), LSIL (low-grade abnormalities), HSIL (high-grade abnormalities); HPV 16/18 (+), HPV 16 or HPV 18 or HPV 16 and 18 positive; HPV 16/18 (-), other 12 HR- HPV types (+), no HPV 16 nor HPV 18.

The performance of the combination of the two assays in identifying CIN2+ cases in ASCUS group was 2/55 (3.6%) when HPV16/18(-)/ p16/ki-67(-), 8/10 (80%) when HPV 16/18(-)/p16/ki-67(+), 0/17 (0%) when HPV 16/18(+)/p16/ki-67(-) and 11/11 (100%) when HPV 16/18(+)/p16/ki-67(+) as shown in Table 4. Further, we compared the colposcopy impression with the pathology report in the ASCUS group and we found that 3 out of 17 (17.6%) CIN2+ cases were evaluated as “Negative”, 15/73 (20.5%) CIN2+ cases as “LSIL” and 3/3 (100%) CIN2+ cases as “HSIL”.

Regarding the LSIL cytology group as shown in Table 4, a total of 52 out of 103 cases were HPV 16/18 positive, of which 40/52 (76.9%) were histologically diagnosed as CIN2+. Concerning the p16/ki-67 method, 60/103 were p16/ki-67 positive of which 58/60 (96.7%) were CIN2+. The performance of the combination of the two assays in identifying CIN2+ cases in the LSIL group as shown in Table 4, was 0/28 (0%) when HPV 16/18(-)/p16/ki-67(-), in 21/23 (91.3%) when HPV 16/18(-)/p16/ki-67(+), 3/15 (20%) when HPV 16/18(+)/p16/ki-67(-) and 37/37 (100%) when HPV 16/18(+)/p16/ki-67(+). Similarly, we compared the colposcopy impression with the pathology report in the LSIL group and we found that 1 out of 7 (14.3%) CIN2+ cases was evaluated as “Negative”, 38/74 (51.4%) as “LSIL” and 22/22 (100%) as “HSIL”.

The diagnostic accuracy of each test for the detection of CIN2+ is presented in Table 5. Dual stain (p16/ki-67) demonstrated superior sensitivity for both cytology groups (ASCUS and LSIL), 90.4% (95% CI 68–98) and 95% (95% CI 85–99) respectively compared to HR-HPV test and Colposcopy. Particularly, the HR-HPV test’s sensitivity was 52.3% (95% CI 30–74) and 65.5% (95% CI 52–77) for the ASCUS and LSIL cytology groups respectively and the Colposcopy’s sensitivity was 14.3% (95% CI 4–37) and 36% (95% CI 24–49) respectively. The p16/ki-67 specificity was 97.2% (95% CI 89–99) and 95.2% (95% CI 83–99) for the ASCUS and LSIL groups, respectively inferior only to colposcopy which showed a specificity of 100% (95% CI 94–100) and 100% (95% CI 89–100), lacking however statistical significance. The HR-HPV test presented the lowest values: 76.4% (95% CI 65–85) and 71.4% (95% CI 55–84) for the ASCUS and LSIL respectively.

**Table 5:** Diagnostic ability of HR-HPV test, colposcopy & p16/ki-67 for the detection of CIN2+.

		HR-HPV test^1^	Colposcopy^2^	p16/ki-67^3^	p-value _1 vs 3_ ^b^	p-value _2 vs 3_ ^b^
**ASCUS**	Sensitivity (CI)^a^	52.3 (30–74)	14.3 (4–37)	90.4 (68–98)	<0.005	<0.005
	Specificity (CI)^a^	76.4 (65–85)	100 (94–100)	97.2 (89–99)	0.001	NS
	PPV (CI)^a^	39.2 (22–60)	100 (31–100)	90.4 (68–98)	<0.005	NS
	NPV (CI)^a^	84.6 (73–92)	80 (70–87)	97.2 (89–99)	0.038	0.027
**LSIL**	Sensitivity (CI)^a^	65.5 (52–77)	36 (24–49)	95 (85–99)	<0.005	<0.005
	Specificity (CI)^a^	71.4 (55–84)	100 (89–100)	95.2 (83–99)	0.013	NS
	PPV (CI)^a^	76.9 (63–87)	100 (81–100)	96.6 (87–99)	0.003	NS
	NPV (CI)^a^	58.8 (44–72)	52 (41–63)	93 (80–98)	<0.005	<0.005

PPV, positive predictive value; NPV, negative predictive value; NS, not significant.

^a^ Values are presented as percentages with 95% confidence intervals (CI).

^b^ Comparisons was performed using the Paired Samples t-test.

The p16/ki-67 Positive Predictive value (PPV) and Negative Predictive Value (NPV), in the ASCUS group were: 90.4% (95% CI 68–98) and 97.2% (95% CI 89–99) as indicated in Table 5. Compared to Colposcopy, the latter with a PPV: 100% (95% CI 31–100) showed a better score but presented lower NPV: 80% (95% CI 70–87). HR-HPV test presented the lowest PPV: 39.2% (95% CI 22–60) and NPV: 84.6% (95% CI 73–92). Similarly, in the LSIL group, p16/ki-67 showed a PPV of 96.6% (95% CI 87–99) and a NPV of 93% (95% CI 80–98). The colposcopy showed higher PPV:100% (95% CI 81–100) but lower NPV:52% (95% CI 41–63). The HR-HPV test presented the lowest PPV 76.9% (95% CI 63–87) and NPV 58.8% (95% CI 44–72) in comparison to the other two methods.

**Fig 6:**
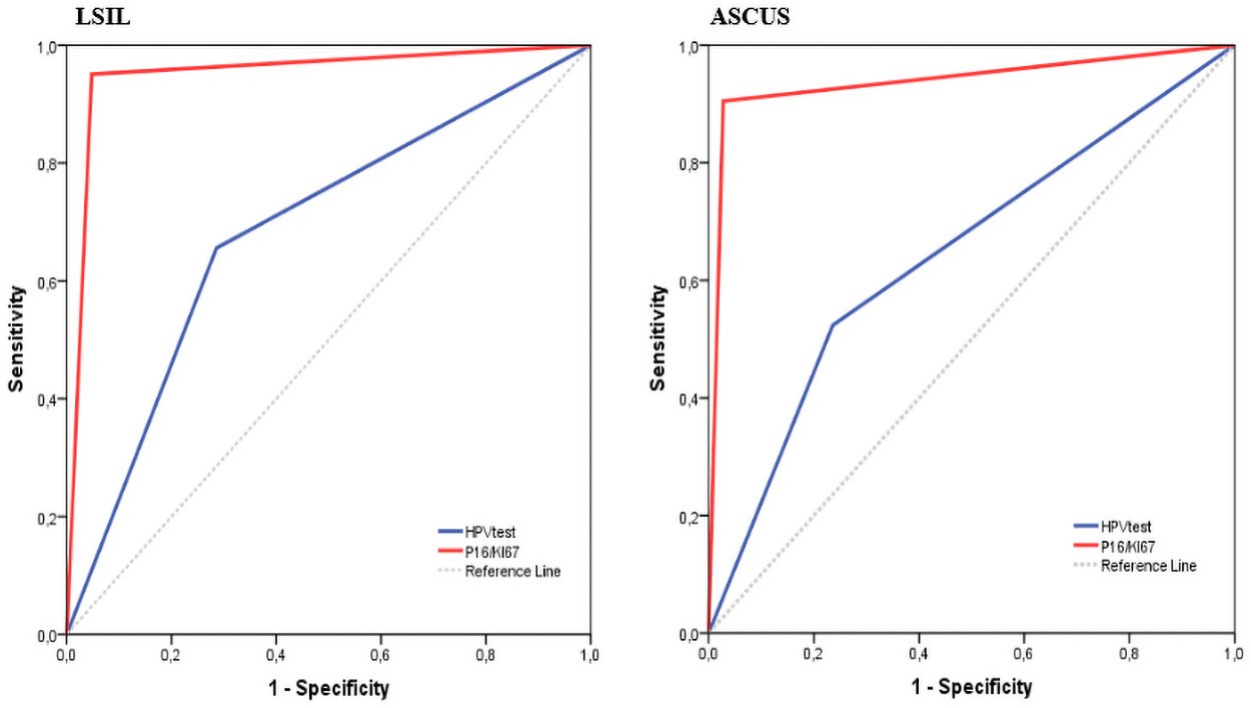
Evaluation of the two methods, HR-HPV test and p16/ki-67 by ROC curve analysis.

The ROC curve analysis of the two methods, HR-HPV test and p16/ki-67, is shown in Fig 6. For the ASCUS group the areas under the curve (AUC) for the HR-HPV test and p16/ki-67 were 0.644 (95% CI 0.503–0.784) and 0.938 (95% CI 0.862–1.000) and for the LSIL group, the AUC of HR-HPV test and p16/ki-67 were 0.685 (95% CI 0.580–0.790) and 0.952 (95% CI 0.903–1.000) respectively.

In order to evaluate the costs of the three different proposed CC screening strategies (Figs 2–4) we estimated, from a healthcare perspective, either in Public Healthcare System or in Private Practice, the total direct medical costs of the procedures during our clinical trial. The total direct medical costs (screening tests and diagnostic methods plus physician’s time during office visit) for our population were calculated as the number of cases in each of the three different screening strategies multiplied by the direct medical costs per case (Table 6). The calculations presented in Table 6 were conducted using the data of the costs in Table 2 and the resource use from the clinical data of our population in Table 4.

**Table 6:** Total direct medical costs, from a healthcare perspective, of the three screening strategies.

Screening Strategies			Public Healthcare System		Private Practice	
		Number of cases*	Direct medical costs per case(€)**	Total direct medical costs of our population(€)	Direct medical costs per case(€)**	Total direct medical costs of our population(€)
**1**^**st**^ **Strategy** Cytology						
**a) HPV test triage**	HPV16/18(+)	80	132,06	10.564,80	537,50	43.000,00
	HPV16/18(-)	116	93,32	10.825,12	345,00	40.020,00
	**Total**	196		21.389,92		83.020,00
**b) p16/ki-67 triage**	p16/ki-67(+)	81	112,06	9.076,86	460,00	37.260,00
	p16/ki-67(-)	115	73,32	8.431,80	267,50	30.762,50
	**Total**	196		17.508,66		68.022,50
**2**^**nd**^ **Strategy** HPV test						
**a) Cytology triage**	ASCUS	93	132,06	12.281,58	537,50	49.987,50
	LSIL	103	132,06	13.602,18	537,50	55.362,50
	**Total**	196		25.883,76		105.350,00
**b) p16/ki-67triage**	p16/ki-67(+)	81	178,74	14.477,94	540,00	43.740,00
	p16/ki-67(-)	115	140,00	16.100,00	347,50	39.962,50
	**Total**	196		30.577,94		83.702,50
**3**^**rd**^ **Strategy** Co-testing (Cytology &HPV test)						
**p16/ki-67 triage**	p16/ki-67(+)	81	192,06	15.556,86	597,50	48.397,50
	p16/ki-67(-)	115	153,32	17.631,80	405,00	46.575,00
	**Total**	196		33.188,66		94.972,50

HPV test, HPV test with 16/18 genotyping; Costs are presented in Euros (€).

*Cases in our population. Clinical data derived from Table 4.

** Direct medical costs of the procedures per woman screened. Data for calculations derived from Table 2.

Regarding the 1^st^ Cytology screening strategy (Fig 2), when triaging with a) HPV test plus 16/18 genotyping the total direct medical costs in our study population would be €21.389,92 in Public Healthcare System and €83.020,00 in Private Practice, whereas with b) p16/ki-67 dual stain triage the costs would be €17.508,66 and €68.022,50 respectively. Concerning the 2^nd^ HPV test screening strategy approach (Fig 3), we calculated that by using a) cytology triage, the direct medical costs for Public Healthcare System and Private Practice would be €25.883,76 and €105.350,00 respectively, while with the b) p16/ki-67 triage test, the total direct costs were estimated as €30.577,94 and €83.702,50. Finally, the total direct medical costs for the 3^rd^ Co-testing (Cytology & HPV test with 16/18 genotyping) screening strategy and triaging with p16/ki-67 dual stain (Fig 4) were €33.188,66 for the Public Healthcare System and €94.972,50 for the Private Practice.

## Discussion

In the present study, we evaluated the clinical performance of p16/ki-67 in comparison to the existing strategy, i.e. HPV testing with colposcopy referrals in a group of Greek population with ASCUS and LSIL cytology. These methods are already applied in the primary screening in most organized health care systems worldwide [5, 7].

Although HPV DNA test appears to be highly sensitive in multiple studies for the detection of precancerous lesions, it lacks specificity. This leads to the detection of a large number of HPV positive women, who are referred to unnecessary colposcopies since HPV DNA test cannot discriminate an active/persistent infection from a transient one [23].

The p16^INK4a^ protein is a cyclin-dependent kinase (CDK) inhibitor that plays an important role in cell cycle regulation. The over expression of E7 oncoprotein, in persistent HR-HPV infections, leads to p16^INK4a^ upregulation which might serve as a biomarker for the detection of cervical precancerous lesions [24, 25]. ki-67 is another marker which is strictly associated with cell proliferation and is expressed normally during cell mitosis [26]. The co-expression of p16 ^INK4a^ and ki-67 in the same cell, does not physically exist in normal cells and this co-expression is considered a valuable marker for cell-cycle deregulation and cell transformation due to HPV infection [27]. Therefore, a positive p16/ki-67 result could indicate a higher-grade dysplasia on the cervix and provide information about CIN2+ lesions [28, 29].

In our study, besides p16/ki-67, we classified HPV positive patients into two categories, as HPV 16/18 (+) and HPV 16/18 (-) according to algorithms for primary HPV screening [30]. In concordance with the well documented bibliography facts, our results show that HPV 16/18 are the most prevalent types for developing CIN2 or higher lesions, in comparison to the other HR-HPV types [31–33].

We also identified the distribution of the 14 HR- HPV types in our Greek population. Briefly, our results are fully consistent with previous studies in Greece, where HPV 16 is the most prevalent type followed by HPV 31 and HPV 51 [34–36]. This observation shows that the distribution of HR-HPV genotypes kept the same pattern despite the population habit alterations and mixed-race interaction due to refugee’s immigration in the country over these years.

In order to evaluate the accuracy of the methods, we compared the p16/ki-67 dual stain with the HPV testing and colposcopy, which are currently proposed by the screening guidelines. In our study, the results showed that p16/ki-67 method had both high sensitivity and specificity as triage test, both in the two cytology groups (ASCUS/LSIL), improving the diagnosis of CIN2+ lesions.

Among women with ASCUS and LSIL, sensitivities of p16/ki-67 dual stain were 90.4% and 95.0% respectively, higher than that of the HR-HPV test (52.3% and 65.5%). Previous studies have shown that p16/ki-67 can be a reliable tool for risk stratification of HPV positive women with mild cervical lesions, reporting high sensitivity values similar to that of HPV test [27, 37–40].

In both ASCUS and LSIL cases, p16/ki-67 showed higher specificity for CIN2+ diagnosis, 97.2% and 95.2% respectively, while no statistical significance was identified when compared to the HPV test (76.4% and 71.4%). Previous studies have shown that p16/ki-67 had higher specificity compared to HPV testing [28, 37–40]. Our data indicated that p16/ki-67 in both cytology groups, had higher PPV: (90.4% / 96.6%) and NPV: (97.2% / 93%), statistically significant compared to HPV test (PPV: 39.2%/ 76.9%, NPV: 84.6% / 58.8%) in accordance to previous studies [27, 37–40].

In our National Health Care System, colposcopy is widely used to detect cervical lesions and biopsy under colposcopy guidance, confirms or excludes CIN2+ lesions. We retrospectively collected colposcopy reports of the population we studied in our Department of Obstetrics & Gynecology which is a referral Department for gynecological cancer. We compared these reports to the histological findings, in order to evaluate the prognostic accuracy of colposcopy.

We observed that in ASCUS and LSIL cases, 38.1% and 65.7% respectively of the histologically confirmed CIN2+ were underestimated by colposcopy and defined as “Negative” or “LSIL”. Whereas, the p16/ki-67 depicted more CIN2+ cases among the two groups (ASCUS: 90.5%, LSIL: 96.7%) and showed clearly higher sensitivity (ASCUS: 90.4%, LSIL: 95%) than Colposcopy (ASCUS: 14.3%, LSIL: 36%) (p> 0.005). On the other hand, there was no statistical significance in specificity between p16/ki-67 and colposcopy impression (95–100%).

There are many factors that could explain this observation such as, training and experience of the clinicians involved, inadequate colposcopy due to lack of objective criteria to classify the lesions macroscopically and co-existing pathologies which tamper with the appearance of the normal mucosa.

Furthermore, we presented the ROC curve to show the diagnostic performance of p16/ki-67 and HPV test. The AUC of p16/ki-67 was greater in both cytology groups (0.938/0.952) compared to the HPV test (0.644/0.685). These results are comparable to those of a recent study were AUC of p16/ki-67 dual stain was (0.778), also significantly higher than that of HPV test (0.503) [41].

Additionally, in both cytology groups, we evaluated the combination of the two methods, HR-HPV test with 16/18 genotyping and p16/ki-67, in order to identify how many cases of CIN2+ will be missed. In the LSIL cases, when HPV16/18 and p16/ki-67 were both positive or negative, no women were misdiagnosed. In case of conflicting results between HPV16/18 and p16/ki-67, taking into consideration the p16/ki-67 positivity alone, we were able to diagnose 91.3% of women with CIN2+ that would otherwise escape diagnosis and only 8.7% were missed. On the other hand, we observed that if only HPV 16/18 was positive we could detect only 20% of CIN2+ and the rest were missed.

Looking into the ASCUS cases, when both HPV16/18 and p16/ki-67 were negative, 3.6% of women with CIN2+ were missed, whereas when HPV 16/18 (+)/p16/ki-67 (+) all CIN2+ were diagnosed. In case of conflicting results between the two tests, when only p16/ki-67 was positive, 80% of women with CIN2+ were accurately diagnosed. Finally, in HPV 16/18 (+)/p16/ki-67(-) cases, no CIN2+ were missed.

Our study showed that p16/ki-67 presented both high sensitivity and specificity values, with only a few CIN2+ being missed. Thus, it appears to be an excellent triage tool for accurate stratification of women with mild cervical lesions. Our results are in line with other articles where the effectiveness of the screening strategies may be improved by the addition of p16/ki-67 dual stain in the triage [42, 43].

Regarding the analysis of the total direct medical costs of the three different CC screening scenarios, from a healthcare perspective, in our clinical trial, in the 1^st^ strategy, Cytology primary screening, the utilization of p16/ki-67 as an optional triage tool has reduced the costs by €3.881,26 in the Public Healthcare System and by €14.997,50 in Private Practice compared to HPV test triage. Additionally, a cost reduction of €21.647,50 in Private Practice was reported in the HPV test-based screening scenario by adapting dual stain in the triage, whereas an elevation of €4.694,18 was shown in the Public Healthcare System in comparison to cytology triage. Finally, the costs for the Co-testing screening strategy were proved to be the highest among all screening scenarios.

It seems that the model Cytology as primary screening strategy and triage with p16/ki-67 dual stain may not only have high clinical and diagnostic impact, avoiding over-diagnosis and over-treatment, but also budgetary impact lowering annual costs for screening and seems to be the most realistic and safe scenario for CC screening.

As for the limitations of our study, the number of cases was small, as well as the fact that the follow-up of the women enrolled was not evaluated and is yet to be accomplished. Further evaluation of the follow-up is necessary to examine the application of the p16/ki-67 biomarker in triaging women with mild cervical lesions, as shown in many previous scientific studies. The role of p16/ki-67 negativity in combination with HR-HPV positivity remains to be fully interpreted and understood.

Summarizing our results, p16/ki-67 dual stain presented both higher sensitivity and specificity, providing good clinical outcomes, while lowering the annual direct medical costs of CC screening. Cytology in combination with p16/ki-67 dual stain is a powerful, safe and affordable tool to triage women with mild cervical lesions, especially in low- and middle-income countries where accurate but also budgetary adaptations of health care services are required.
